# Preparedness and performance in pediatric assessment: linking OSCE, written exams, and training exposure in a two-year comparative study of medical students

**DOI:** 10.1186/s12909-026-08999-x

**Published:** 2026-03-18

**Authors:** Anaïs Lemoine, Nada Sabourdin, Maxens Decavèle, Romain Guedj, Alain Carrié, Marie-Christine Renaud, Jean-Philippe Foy, Manon Allaire, Antoine Monsel, Jessica Taytard

**Affiliations:** 1 Pediatric Nutrition and Gastroenterology Department, AP-HP, Trousseau Hospital, Sorbonne Université, Paris, France; 2 INSERM, Nutrition and obesities: systemic approaches, Nutriomics, Sorbonne Université, Paris, France; 3 OSCE Research Group, Sorbonne Université, Paris, France; 4 Department of Anesthesiology and Intensive Care, AP-HP, Trousseau Hospital, GRC 29, Sorbonne Université, Paris, France; 5 Pharmacologie et Evaluation des Thérapeutiques chez l’Enfant et la Femme Enceinte, University of Paris, Paris, France; 6 Service de Médecine Intensive - Réanimation (Département R3S), AP-HP, La Pitié-Salpêtrière Hospital, Sorbonne Université, Paris, France; 7 INSERM, UMRS1158 Neurophysiologie Respiratoire Expérimentale et Clinique, Sorbonne Université, Paris, France; 8 Paediatric Emergency Department, AP-HP, Trousseau Hospital, Sorbonne Université, Paris, France; 9 INSERM UMR 1153, Sorbonne Université, Paris, France; 10 Faculty of Medicine Pitié-Salpêtrière, INSERM UMRS 1166, Sorbonne Université, Paris, France; 11 Department of Maxillo-Facial Surgery, AP-HP, La Pitié-Salpêtrière Hospital, Paris, France; 12 INSERM UMRS 938, Centre de Recherche de Saint Antoine, Team Cancer Biology and Therapeutics, Sorbonne Université, Paris, France; 13 Department of hepato-gastroenterology, AP-HP, La Pitié-Salpêtrière Hospital, Sorbonne Université, Paris, France; 14 Team Proliferation, Stress and Liver Physiopathology, INSERM UMRS 1138, Centre de Recherche des Cordeliers, Paris, France; 15 Department of Anesthesiology and Critical Care, Multidisciplinary Intensive Care Unit, La Pitié-Salpêtrière Hospital, Sorbonne Université, Paris, France; 16 INSERM UMRS 959, Immunology-Immunopathology-Immunotherapy (I3), Paris, France; 17 Biotherapy (CIC-BTi) and Inflammation-Immunopathology-Biotherapy Department (DHU i2B), APHP-Pitié-Salpêtrière Hospital, Paris, France; 18 INSERM UMRS 938, Centre de Recherche Saint-Antoine, Sorbonne Université, Paris, 75012 France; 19 Pulmonary function testing and Sleep Department, AP-HP, Hôpital Trousseau, Paris, France; 20 INSERM UMRS 1158 Neurophysiologie Respiratoire Expérimentale et Clinique, Sorbonne Université, Paris, France

**Keywords:** OSCE, Pediatrics, Competencies, Evaluation, Satisfaction

## Abstract

**Background:**

Objective structured clinical examinations (OSCEs) have recently been integrated as one of the academic assessment tools for French undergraduate medical students. Data are scarce regarding the correlation between OSCE (practical skills) and written examination (pure knowledge) scores, especially in pediatrics. We mainly sought to 1) measure the correlation between pediatric OSCE and written examination scores in undergraduate medical students, and 2) assess student perceptions of the OSCE experience.

**Methods:**

This was a single-center retrospective analysis of two consecutive classes of fifth-year medical students, university years 2021 (class 1, n=345) and 2022 (class 2, n=492). Both raw and weighted (individual student score/mean class score) formative OSCE and written examination scores were collected, and correlations were tested (Spearman rank tests). Student perceptions regarding OSCE preparation and performance were evaluated through an anonymous online survey (n=289 answers).

**Results:**

We evidenced significant but weak correlations between OSCE and written examination scores (weighted scores, Class 1: rho=0.29, p<0.001, Class 2: rho=0.21, p<0.001). Only 45.3% of students who scored above the 66th percentile in written examinations also scored above the 66th percentile in the OSCEs. Students who had prior OSCE exposure during informal training while on placements reported feeling better prepared for this type of assessment.

**Conclusions:**

Weak correlation existed between pediatric formative OSCE scores and written examination performance. Exposing students regularly to formative OSCEs made them feel better prepared, with a likely positive impact on anxiety related to this mode of assessment.

**Trial registration:**

N/A

**Supplementary Information:**

The online version contains supplementary material available at 10.1186/s12909-026-08999-x.

## Background

Developed in the 1970s by Harden et al. [1, 2]., Objective Structured Clinical Examinations (OSCEs) have been widely adopted internationally [3] and are considered the gold standard for evaluating clinical competencies in medical education [4]. These examinations consist of timed stations where students demonstrate their clinical skills through standardized scenarios with simulated patients under direct observation [5].

In France, the second cycle of medical studies recently underwent a reform (R2C), with a decree published in 2021, to be implemented in the academic year 2023–2024 [6]. Since 2023, national summative OSCEs (nOSCEs) have become mandatory in French medical education to allow sixth-year medical students to progress to post-graduate medical education (i.e. third cycle of medical studies) after successfully completing summative national digital exams. These nOSCEs account for 30% of the total score in the national matching procedure, which determines the choice of specialty and location for the third cycle.

Since the Ministry of Higher Education, Research, and Innovation announced the second-cycle reform, educators have gradually been trained to create OSCEs station scenarios and standardized scoring grids. Students are also increasingly being trained in this new assessment modality during their hospital placements. In preparation for this national requirement, pediatric formative OSCEs (pedOSCEs) have been implemented for fifth-year medical students since the academic year 2021–2022, complementing existing written formative examinations that assess theoretical knowledge. While OSCE training opportunities are increasingly available during pediatric hospital rotations, the frequency and format of this training vary across different clinical placements.

OSCEs assess different skills (medical knowledge, and analysis and reasoning skills, but also real-time patient care skills, interpersonal communication skills, and technical or procedural skills) from written exams (pure knowledge) [7–9] and data are scarce regarding the correlation between OSCE and written examination scores [10, 11], especially in pediatrics [4, 7]. Our research hypothesis was that there is a significant positive correlation between students’ performance in both assessment modes. We also postulated that increased OSCE training during undergraduate medical education would enhance student performance in OSCEs. This study aimed to (1) analyze the correlation between scores in formative pedOSCEs and written examinations, (2) compare pedOSCE performance across two consecutive academic years, and (3) assess student perceptions of formative pedOSCEs.

## Methods

**Fig. 1:**
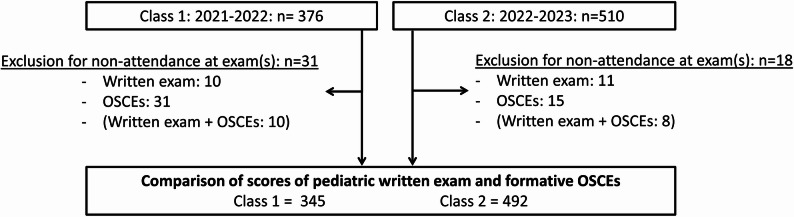
Flow chart

This single-center retrospective study was conducted at the Sorbonne University Faculty of Medicine in Paris, France. The subjects included were fifth-year medical students undergoing a pediatric rotation, during the academic years 2021–2022 (Class 1, unaffected by the reform) and 2022–2023 (Class 2, after the reform). Students in Class 2 were exposed to OSCE training (meaning informal OSCE proposed by some supervisors during their pediatric and non-pediatric rotations) during academic year 2021–2022, which is why we assumed that the students in Class 2 were more trained for OSCE than those in Class 1. Students who did not take the pediatric written exam and/or pedOSCEs in 2021–2022 (Class 1) or 2022–2023 (Class 2) were excluded from the analysis (Fig. 1). The pediatric written exam consisted of 50 multiple-choice questions (MCQs) or single-answer questions which were drafted and validated by several experienced local faculty members. The questions were in the form of progressive clinical cases and individual theoretical questions, The exam was taken over the course of one hour and was graded out of 20. There was one or two sessions per year to assess the entire student class (supplementary figure). If students did not achieve the required average, they had to retake the pediatric written exam during a resit session. After two failed attempts, they had to repeat the year.The formative pedOSCE session consisted of two 8-minute stations (reading the case description and clinical scenario, followed by one minute of individual debriefing), graded out of 20 in total. PedOSCEs covered various topics based on an official and national list of “initial situations” and “learning areas” [12–14] related to diagnosis, prevention, management, clinical examination skills, analysis of additional laboratory or radiological tests, and prescription skills for example. Up to 60% of the score evaluated general objectives, such as the ability to be a clinician, communicator, reflective practitioner, public health actor and/or if students demonstrated ethical skills with a patient-centered approach. As for written exams, several experienced local faculty members validated the OSCEs grid before assessing the students. A briefing was held for the assessors to standardize student grading. Two faculty members played the roles of standardized parents or healthcare professionals, or assessors at each station. Eight sessions per year (two sessions per hospital rotation) were required to assess the whole student class (supplementary figure). The scores from these formative pediatric OSCEs were only given to the students to help them assess their performance, but were not penalizing even if below average.

All topics and questions in the formative assessments were aligned with the national pediatrics curriculum (official national program of pediatrics curriculum in a reference textbook from the National College of University Pediatricians and the National College of Hospital and University Pediatric Surgeons) and form part of the expected learning outcomes at the end of the second cycle of medical studies [12–14].

Each student only underwent one formative written exam and one formative pedOSCE in pediatrics per year. Each score in the written exam and pedOSCE was expressed both as a raw value and as a weighted value, which corresponded to the raw score divided by the class’s mean score for that session. The weighted value of the score was used to evaluate how a student performed compared to other students who did not take the same exam.

Furthermore, students from Classes 1 and 2 were encouraged to complete an anonymous online questionnaire (Google Forms^®^), without knowing their scores, to assess their satisfaction with, and their level of preparation for the formative pedOSCEs, taking into account their past experience with formative and training OSCEs they had completed during their pediatric and non-pediatric rotations (supplementary file). Training OSCEs consist of informal OSCE sessions during the placements, in which students sometimes participate as standardized participants or patients. Debriefing in training OSCEs, consists of showing students the grading scale and their scores, and providing them constructive feedback, either individually or in groups. It is usually longer and more personalized than for formative OSCEs. The survey link was sent only once in June 2022 to Class 1 and multiple times during the 2022–2023 academic year to Class 2, after each formative pedOSCE session. Responses to the online questionnaire were last collected on July 31, 2023. Responses took the form of a five-point Likert scale, binary (yes/no) responses, or open comments.

### Statistical analysis

Data were collected using Microsoft Excel (version 1910). Statistical analyses were conducted using GraphPad Prism 9 (version 9.5.1, GraphPad Software, LLC). Continuous variables were reported as median and interquartile intervals or extremes, and categorical variables were reported as frequencies (%). Paired Wilcoxon tests were used to compare scores for each student between exams (non-Gaussian distribution of scores according to a Shapiro-Wilk normality test). Spearman rank correlations were used to assess the relationship between pedOSCE and written exam scores in each class (rho). Mann-Whitney tests were used to compare scores between the two classes. Kruskal-Wallis tests compared scores between all exam sessions (three and 16 different sessions for written exams and pedOSCEs respectively for the whole class). The weighted written exam and pedOSCE scores were converted into percentiles. The Sankey diagram was designed from the contingency table of the weighted score distribution (above the 66th percentile, below the 33rd percentile, average [33rd-66th] percentile) thanks to the website https://SankeyMATIC.com. The distribution of quantitative variables in different groups was evaluated using a contingency test or the chi-squared test, with grouping if the observed frequencies were insufficient or with Yates’s correction if the calculated frequencies were insufficient. The significance threshold was set at 0.05.

The study was conducted in accordance with the Declaration of Helsinki, and was approved by the Ethics Research Committee of the French Society of Pediatrics in June 2023 (Opinion no. CERSFP-2023-151). Students were given written information about their right to object to the collection of their data within one month. As the study was considered “research not involving human subjects” under French and European legislation, data from students who did not object were included in the analysis.

## Results

### Part 1: scores

Figure 1 represents the study flow chart. Class 1 was composed of 376 students and Class 2 of 510 students. After excluding students who did not participate in the exams, we included 345 students from Class 1 and 492 from Class 2 in the analyses.

In Class 1, the raw pedOSCE scores were significantly lower than the written exam scores in the paired analysis (Table 1). There was only a weak correlation between the raw pedOSCE and written exam scores (rho = 0.27; 95% confidence interval [CI] 0.17 to 0.37; *p* < 0.001). In Class 2, the raw pedOSCE scores were also significantly lower than the written exam scores in the paired analysis (Table 1) and were very weakly correlated (rho = 0.11; 95% CI 0.02 to 0.20; *p* = 0.01). After weighting the scores to the mean score for each exam session, the student scores no longer differed between the two exam modes (Table 1), although they were still correlated (Class 1: rho = 0.29, 95% CI 0.19–0.39, *p* < 0.001; Class 2: rho = 0.21, 95% CI 0.12–0.30, *p* < 0.001).

**Table 1:** Scores to pediatric formative OSCEs and written exams

	OSCEs	Written exams	Spearman correlation	*P*-value^*#*^
Class 1 (n=345)				
Raw scores	13.3 (6.1-18.9)	13.8 (9.3-18.8)	ρ=0.27, 95%CI [0.17-0.37], p<0.001	<0.001
Weighed scores	1.02 (0.47-1.44)	1.00 (0.66-1.42)	ρ=0.29, 95%CI [0.19-0.39], p<0.001	0.8
Class 2 (n=492)				
Raw scores	12.5 (4.5-18.5)	16.1 (8.9-19.0)	ρ=0.11, 95%CI [0.02-0.20], p=0.01	
Weighed scores	1.02 (0.34-1.44)	1.03 (0.56-1.21)	ρ=0.21, 95%CI [0.12-0.30], p<0.001	<0.001
*P*-value^*¤*^				0.8
Raw scores	0.06	<0.001		
Weighted scores	0.7	0.08		

Scores expressed as medians and extremes

*OSCEs* Objective Structured Clinical Examinations, 95%*IC* 95% Confidence interval

^#^Wilcoxon tests for intra-class comparison of OSCEs vs written exam

^¤^Mann-Whitney tests for comparison between classes

The raw scores for the pediatric written exam were significantly higher for students in Class 2 than for students in Class 1 (Table 1).

Raw scores also differed significantly between each exam session (Kruskal-Wallis test: written exam: *p* < 0.001; pedOSCEs: *p* < 0.001). Weighted scores, on the other hand, showed no significant differences between the two classes (Table 1) or between different exam sessions (written exam: *p* = 0.21; pedOSCEs: *p* > 0.99). There was no difference in the weighted pedOSCE scores depending on whether the students had taken the written exam before or after the pedOSCEs (*p* = 0.69).

**Fig. 2:**
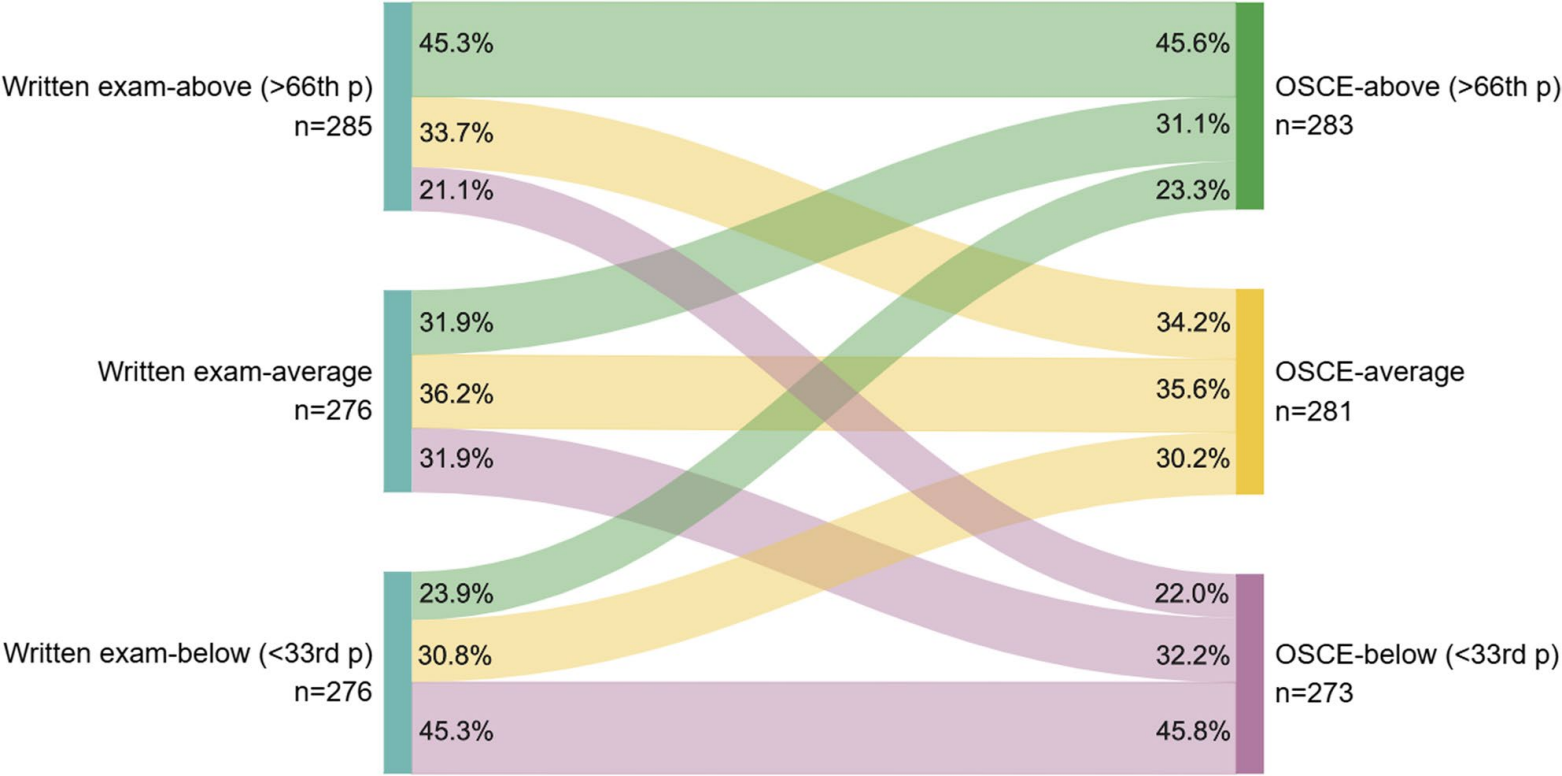
Sankey diagram of written exam and OSCE scores according to percentiles of weighted scores

Across both classes, only 45.3% of students who scored above the 66th percentile in written examinations also scored above the 66th percentile in the pedOSCEs; the opposite was also true for the lowest scores in the pedOSCEs (45.3%) and written exam (below the 33rd percentile) (chi² test, *p* < 0.0001) (Fig. 2).

### Part 2: questionnaire

A total of 289 responses were collected through the online questionnaire, with 22.5% coming from Class 1 (*n* = 65) and 77.5% from Class 2 (*n* = 224). The median age was 23 years (range 22–24, extremes 21–38).

Students’ perceptions of the pedOSCEs were not different between the two classes regarding the “difficulty” of the test, the “stress” related to the evaluation and whether they considered the results to be representative of their learning during their pediatric placement (Table 2). Students in Class 1 were generally more satisfied with their performance than those in Class 2 (*p* = 0.03), with a significant majority of students (52.3% vs. 36.2%, respectively, *p* = 0.02) reporting being rather or very satisfied with their performance (Table 2).

**Table 2:** Students’ perception of formative OSCEs

	Class 1 n=65		Class 2 n=224	*P*-value
Pediatric formative OSCEs				
*“How do you rate the difficulty of the pediatric OSCE you just completed?”*				
Overall answer	3.0; 3.0 (2.5-3.0)		3.1; 3.0 (3.0-3.0)	0.08
1-2 (1: very easy)	16 (24.6%)		30 (13.4%)	0.09
3	36 (55.4%)		139 (62.1%)	
4-5 (5: very difficult)	13 (20.0%)		55 (24.6%)	
*“Did you find this assessment stressful?”*				
Overall answer	3.0; 3.0 (2.0-4.0)		3.1; 3.0 (2.0-4.0)	0.83
1: not stressful	7 (10.8%)		22 (9.8%)	0.56
2	13 (20.0%)		48 (21.4%)	
3	20 (30.8%)		70 (31.3%)	
4	22 (33.8%)		60 (26.8%)	
5: very stressful	3 (4.6%)		24 (10.7%)	
*“Were you satisfied with your performance?”*				
Overall answer	3.3; 4.0 (3.0-4.0)		3.1; 3.0 (2.0-4.0)	**0.03**
1: very dissatisfied	3 (4.6%)		18 (8.0%)	0.11
2	8 (12.3%)		42 (18.8%)	
3	20 (30.8%)		83 (37.1%)	
4	32 (49.2%)		70 (31.3%)	
5: very satisfied	2 (3.1%)		11 (4.9%)	
*“Do you think the test result reflects the skills you acquired during the rotation?”*				
Overall answer	2.6; 3.0 (2.0-3.0)		2.6; 3.0 (2.0-3.0)	0.95
1: not representative at all	13 (20.0%)		37 (16.5%)	0.11
2	14 (21.5%)		69 (30.8%)	
3	27 (41.5%)		66 (29.5%)	
4	6 (9.2%)		41 (18.3%)	
5: very representative	5 (7.7%)		11 (4.9%)	
Formative OSCEs in general				
*“Have you ever been assessed throughOSCEs during your non-pediatric rotation?”*				
Positive answer	62 (95.4%)	217 (96.9%)		0.85
*“Have you ever been assessed through OSCEs during your pediatric rotation?”*				
Positive answer	16 (24.6%)	71 (31.7%)		0.27
*“Do you feel prepared for this type of assessment?”*				
Overall answer	2.8; 3.0 (2.0-4.0)	2.9; 3.0 (2.0-4.0)		0.52
1: not well prepared at all	9 (13.8%)	21 (9.4%)		0.88
2	12 (18.5%)	41 (18.3%)		
3	27 (41.5%)	101 (45.1%)		
4	15 (23.1%)	54 (24.1%)		
5: very well prepared	2 (3.1%)	7 (3.1%)		
*“Do you think this is a reliable method to assess your theoretical skills?”*				
Overall answer	2.8; 3.0 (2.0-4.0)	2.7; 3.0 (2.0-4.0)		0.84
1: not reliable at all	8 (12.3%)	29 (12.9%)		0.91
2	18 (27.7%)	67 (29.9%)		
3	22 (33.8%)	70 (31.3%)		
4	16 (24.6%)	50 (22.3%)		
5: very reliable	1 (1.5%)	8 (3.6%)		
*“Do you think it is a reliable method to assess your practical skills?”*				
Overall answer	3.3; 4.0 (2.5-4.0)	3.1; 3.0 (2.0-4.0)		0.13
1: not reliable at all	6 (9.2%)	26 (11.6%)		0.43
2	10 (15.4%)	42 (18.8%)		
3	13 (20.0%)	62 (27.7%)		
4	27 (41.5%)	68 (30.4%)		
5: very reliable	9 (13.8%)	26 (11.6%)		
*“Are you satisfied with this type of assessment?”*				
Overall answer	3.4; 3.0 (3.0-4.0)	3.3; 3.0 (3.0-4.0)		0.95
1: very satisfied	2 (3.1%)	12 (5.4%)		0.86
2	9 (13.8%)	35 (15.6%)		
3	25 (38.5%)	74 (33.0%)		
4	21 (32.3%)	70 (31.3%)		
5: very dissatisfied	8 (12.3%)	33 (14.7%)		
*“Do you believe that this assessment method can help you progress?”*				
Overall answer	3.9; 3.0 (3.0-5.0)	4.0; 4.0 (4.0-5.0)		0.65
1: absolutely not	2 (3.1%)	9 (4.0%)		0.94
2	4 (6.2%)	14 (6.3%)		
3	12 (18.5%)	32 (14.3%)		
4	25 (38.5%)	87 (38.8%)		
5: absolutely	22 (33.8%)	82 (36.6%)		

Overall answers expressed as mean, median and interquartile range (IQR); Mann-Whitney test. Detailed responses (Yes/No and Lickert scale from 1 to 5) expressed in counts and proportions per class; Chi-squared test

*OSCE* Objective structured clinical examination

A large number of students in both classes (*n* = 133/289, 46.0%) considered that the formative pedOSCEs were not representative (responses 1 and 2: “not at all representative” and “not very representative”) of their learning during the pediatric rotation (Table 2).

Almost all students (*n* = 279/289, 96.5%) had already experienced OSCEs during previous non-pediatric rotations (Table 2). A quarter of the students in Class 1 had been trained with previous training OSCEs during their pediatric rotations and nearly a third in Class 2 (Table 2).

**Fig. 3:**
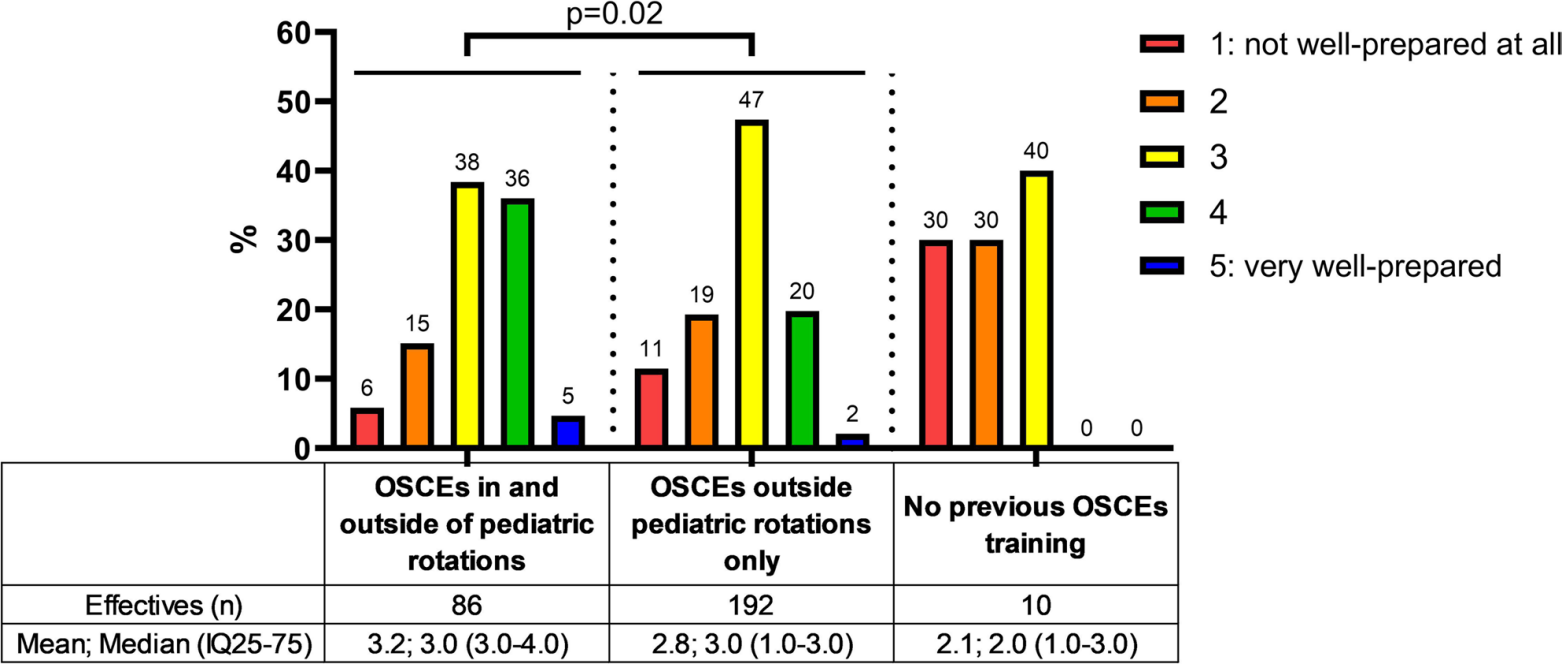
Distribution of responses to the question regarding the feeling of being prepared for OSCEs, based on past experience with OSCEs. Legend: Values in graph expressed as percentages based on responses. Significant difference in response distributions between the "OSCEs in and outside pediatric rotations" group and the "OSCEs outside pediatric rotations only" group (Chi-squared test, *p*-value=0.02). Contingency test not performed for the "No previous OSCEs training” group due to small sample size (*n*=10). OSCEs: Objective Structured Clinical Examinations

The feeling of being prepared for pedOSCEs varied significantly depending on the training they had previously received (Fig. 3). Through the both classes, 66.4% of students had participated in OSCEs outside of pediatrics only, 29.8% of students had participated in OSCEs during both pediatric and non-pediatric rotations, and a minority (3.5%) reported never having undergone OSCEs before the formative pedOSCEs. Students who had experience with OSCEs in both pediatrics and other fields felt the most prepared compared to the other two categories (Kruskal-Wallis test: *p* < 0.001; pediatric and non-pediatric OSCEs versus no prior OSCEs: adjusted p-value < 0.005; pediatric and non-pediatric OSCEs versus non-pediatric OSCEs only: adjusted p-value < 0.01) (Fig. 3).

Satisfaction with OSCEs in general, specific satisfaction following the formative pedOSCEs, and the stress experienced during the formative pedOSCEs did not differ based on the students’ training levels (Table 2). Level of satisfaction with performance during the pedOSCEs (5-point scale, 5 = very satisfied), and level of stress experienced during assessment (5-point scale, 5 = very stressful) were negatively correlated (rho=-0.35, *p* < 0.001).

One third (*n* = 97, 33.6%) of the respondents gave us free comments. The main free comments were generally favorable towards the initiative of pediatric teachers to provide formative pedOSCEs at the end of the pediatric rotation (*n* = 44, 45.4% of acknowledgements). Students wished for more than two stations and for more training OSCEs during rotations (*n* = 23, 23.7%). Students also appreciated the one-minute debriefing at the end of each pedOSCE station but asked either for a longer debriefing or to see the grid, and they wanted to receive their scores quickly after the assessment (*n* = 20, 20.6%). Some students expressed negative concerns and/or suggested ways to improve (*n* = 34, 35.1%), in particular regarding student instructions or pedOSCE contents (*n* = 15), the objectiveness of the evaluations and/or concern about nOSCEs (*n* = 14), and difficulties associated with role-playing (*n* = 7).

## Discussion

This study showed that (1) formative pedOSCEs were weakly correlated with the pediatric written examination taken during the same year for two successive classes of medical students; (2) more than half of the students who scored higher in the written exams were average or below average in the pedOSCE; (3) students who had received training during both their pediatric and non-pediatric rotations felt better prepared for this mode of assessment.

As reported by Lebdai et al. [15] and Nguyen et al. [11]. in two other French medical universities, raw OSCE scores were weakly to moderately correlated with performance in written clinical case assessments (*r* = 0.3 and *r* = 0.39, respectively, compared to rho = 0.27 in Class 1 and rho = 0.11 in Class 2). In our study, correlations were slightly stronger after weighting the scores based on the class average. Since the scores were not equivalent across the different examination sessions, normalizing the scores by weighting them to the class average allowed for a more meaningful comparison of students’ performance relative to their peers [11]. One of the reasons that could explain the weaker correlation was the limited number of stations during our formative pedOSCEs (*n* = 2) compared to previous publications (*n* = 7 at Angers University for Lebdai et al. [15] ; *n* = 3 to 5 at Dijon University for Nguyen et al. [11]). In addition, we showed that for more than half the students, a high score in the written exam was not a predictor of a high score in the pedOSCEs. Indeed, OSCEs assess different skills (practical) than written exams (theorical) and this result highlights the complementary nature of these two assessment tools while also providing a strong educational argument for OSCE implementation in medical studies. Daniel et al. reviewed 377 articles and rated each assessment method in terms of its ability to assess different components of clinical reasoning. They scored assessment method on a 2-point scale (0.0-0.5: poor; 0.6–1.0: average; 1.1–1.5: good; 1.6–2.0: very good). Compared to clinical cases with MCQs, they reported that OSCEs more reliably evaluate the ability to gather information on a clinical scenario (2 versus 0.9), formulate diagnostic hypotheses (1.3 versus 0.3), identify problems in the case (1.3 versus 0), consider differential diagnoses (1.8 versus 0.6), and justify the final diagnosis (1.3 versus 0). Regarding reaching a diagnosis, and the required management and treatment for the case, clinical cases with MCQs were slightly superior to OSCEs (1.9 versus 1.7 and 1.8 versus 1.7, respectively) [7]. Therefore, OSCEs assess broader medical competencies than written clinical cases and are less theoretical.

The more students had participated in OSCEs during their rotations, the more prepared they felt for this form of assessment. Nguyen et al. had also been demonstrated that involving students as standardized patients significantly improved their performance in OSCEs as evaluated students [16]. Although the level of stress, perceived difficulty, and communication skills were similar between students who played the role of standardized patients and those who did not, students who had experienced this role reported being better prepared for the assessment day [16]. Students also seem to appreciate when standardized patients are simulated by students from older classes rather than by peers from their own class, which boosts their confidence [17]. At our faculty level, we consider probably relevant that all hospital rotations, regardless of the specialty, offer regular formative training OSCEs to prepare students for summative OSCEs. According to the literature [16, 17], it also seems interesting to involve students as standardized patients, from both a training and organizational perspective, as the number of people required to implement OSCEs is substantial. Furthermore, OSCEs are one of the most anxiety-provoking assessments [18] compared with written examinations [19]. In dental education, anxiety during OSCEs seems to be associated with the level of OSCE preparation, but not with the OSCE score [19]. In our study, the students reported being equally stressed and in the same proportions in Class 1 and 2. It would be interesting to objectively assess students’ anxiety with a specific questionnaire, feeling of readiness for OSCEs on the day of evaluation, and self-evaluation of performance, based on their training level beforehand.

Our students told us that they appreciated and requested the personalized debriefing at the end of each station; one or two minutes were dedicated to this. Debriefing can be done in various ways: either individually and personalized, immediate or delayed, or collectively, more general, and delayed over time. For example, in a university in Malaysia, fourth-year medical students reported feeling more comfortable with a detailed, written debriefing (including detailed scores, scoring sheets, and comments from the examiner) than with a one-on-one debriefing [20]. They felt that this type of debriefing improved their performance, especially when it included a scoring sheet [20]. Their educators were supportive of both techniques [20]. If written feedback is provided to a student, it should be specific and constructive, thanks to a structured format, for example [21]. When students acted as standardized patients or evaluators, the assessed students also appreciated the individual peer debriefing, especially when it was done by students from older classes rather than by peers from their own class [17, 22].

Many students (46.0%) at our university felt that the formative pedOSCEs they had taken were not representative of the skills acquired during their rotation. This may have been due to the limited number of stations per session (only two) in these formative pedOSCEs and the fact that the vast majority of pediatric rotation sites at Sorbonne University are in highly specialized hospital departments. However, the items addressed during the pedOSCEs always corresponded to the program of the last edition of the reference book from the National College of University Pediatricians and the National College of Hospital and University Pediatric Surgeons published in 2021. To increase the number of stations per session, and given the limited number of available teachers, it would be advisable to involve students from different levels as standardized patients after appropriate training.

This study had several limitations. Firstly, the multiplicity of examination sessions made it difficult to compare raw scores between students and classes because of non-equivalent examination difficulties, which is why we also utilized weighted scores for the analyses. There may have been inequity in the scoring of the pedOSCEs by evaluators, with a subjective component in some parts of the scoring sheet, and with some examiners being more or less lenient. Efforts are currently being made to make the scoring sheets as objective as possible and ensure that all educators are trained in this mode of assessment. The online questionnaire did not collect comprehensive responses from each class, especially Class 1 (the questionnaire was sent late in the academic year, explaining the low rate of participation, and the students were not yet well prepared for OSCEs and were not affected by the second-cycle reform). No correlation could be made between the pedOSCE score, the training level of each student and their personal satisfaction following their evaluation, as this information was not available because of the anonymity of the questionnaire.

In terms of current and future prospects, starting in the 2023–2024 academic year, an individual one- to two-minute debriefing is now systematically offered to students at the end of each station, along with a score report at the closing of the pediatric rotation. Additionally, an opportunity for students to interact with the pedOSCE’s author via video conferencing could also be provided. All formative pedOSCEs have been made available to educators to create a bank of pedOSCEs. Educators receive enhanced training in creating OSCEs, with the same requirements as for faculty OSCEs. In the coming years, all pediatric rotations will offer training OSCEs, and efforts will be made to increase the number of stations in formative pedOSCEs at the end of the pediatric rotation by involving younger students as standardized patients for example. While the formative value of OSCEs no longer seems to be called into question, the relevance of ranking students in a national competition remains to be demonstrated.

## Conclusions

Formative pedOSCEs served as a weakly correlated assessment method complementary to written clinical case evaluations. Exposing students regularly to formative OSCEs made them feel better prepared, with a likely positive impact on anxiety related to this mode of assessment.

## Supplementary Information

Supplementary Material 1.

Supplementary Material 2. Supplementary figure: chronology of formative and summative examinations by class, according to the second-cycle reform (R2C).

## Data Availability

Deidentified individual participant data (including data dictionaries) will be made available, upon publication to researchers who provide a methodologically sound proposal for use in achieving the goals of the approved proposal. Proposals should be submitted to the corresponding author.

## Abbreviations

OSCE: Objective structured clinical examination
pedOSCE: Pediatric objective structured clinical examination
nOSCE: National objective structured clinical examination
