# Supplementary material for: Preparedness and performance in pediatric assessment: linking OSCE, written exams, and training exposure in a two-year comparative study of medical students
Source: BMC Med Educ. 2026 Mar 18;26:676. doi: 10.1186/s12909-026-08999-x (PMC13112695; doi:10.1186/s12909-026-08999-x)
Supplement: Supplementary file 1 — Supplementary Material 1. [file 12909_2026_8999_MOESM1_ESM.docx]

**Pediatric OSCEs – STUDENTS**
Satisfaction questionnaire on the conduct of the Pediatric OSCEs

**Age***: ….

**Non-medical curriculum***:
□ Yes □ No
If yes, specify*: ….

**OSCE Procedure**

**How do you rate the difficulty of the pediatric OSCE you just completed?***
Very easy: 1 > Very difficult: 5
□ 1 □ 2 □ 3 □ 4 □ 5

**Did you find the evaluation stressful?***
Not stressful: 1 > Very stressful: 5
□ 1 □ 2 □ 3 □ 4 □ 5

**Were you satisfied with your performance?***
Very dissatisfied: 1 > Very satisfied: 5
□ 1 □ 2 □ 3 □ 4 □ 5

**Do you think the test result reflects the skills you acquired during the rotation?***
Not representative at all: 1 > Very representative: 5
□ 1 □ 2 □ 3 □ 4 □ 5

**General**

**Have you ever been assessed through OSCEs during your non-pediatric rotations?***
□ Yes □ No
If yes, specify (number of evaluations, number of stations)*: …….

**Have you ever been assessed through OSCEs during your pediatrics rotation?***
□ Yes □ No
If yes, specify (number of evaluations, number of stations)*: …….

**Do you feel prepared for this type of assessment?***
Not prepared at all: 1 > Very well prepared: 5
□ 1 □ 2 □ 3 □ 4 □ 5

**Do you think this is a reliable method to assess your theoretical skills?***
Not reliable at all: 1 > Very reliable: 5
□ 1 □ 2 □ 3 □ 4 □ 5

**Do you think this is a reliable method to assess your practical skills?***
Not reliable at all: 1 > Very reliable: 5
□ 1 □ 2 □ 3 □ 4 □ 5

**Are you satisfied with this type of assessment?***
Very dissatisfied: 1 > Very satisfied: 5
□ 1 □ 2 □ 3 □ 4 □ 5

**Do you believe that this assessment method can help you progress?***
Absolutely not: 1 > Absolutely: 5
□ 1 □ 2 □ 3 □ 4 □ 5

**Comments and Suggestions**

**Comments and suggestions to improve the Pediatric OSCEs**
(Open-ended response)

**Are there any practical skills you would like to see addressed through short videos?**
(e.g., preparing a baby bottle, using a spacer for inhalation, etc.)
(Open-ended response)
