# Supplementary figures and images for: Preparedness and performance in pediatric assessment: linking OSCE, written exams, and training exposure in a two-year comparative study of medical students
Source: BMC Med Educ. 2026 Mar 18;26:676. doi: 10.1186/s12909-026-08999-x (PMC13112695; doi:10.1186/s12909-026-08999-x)

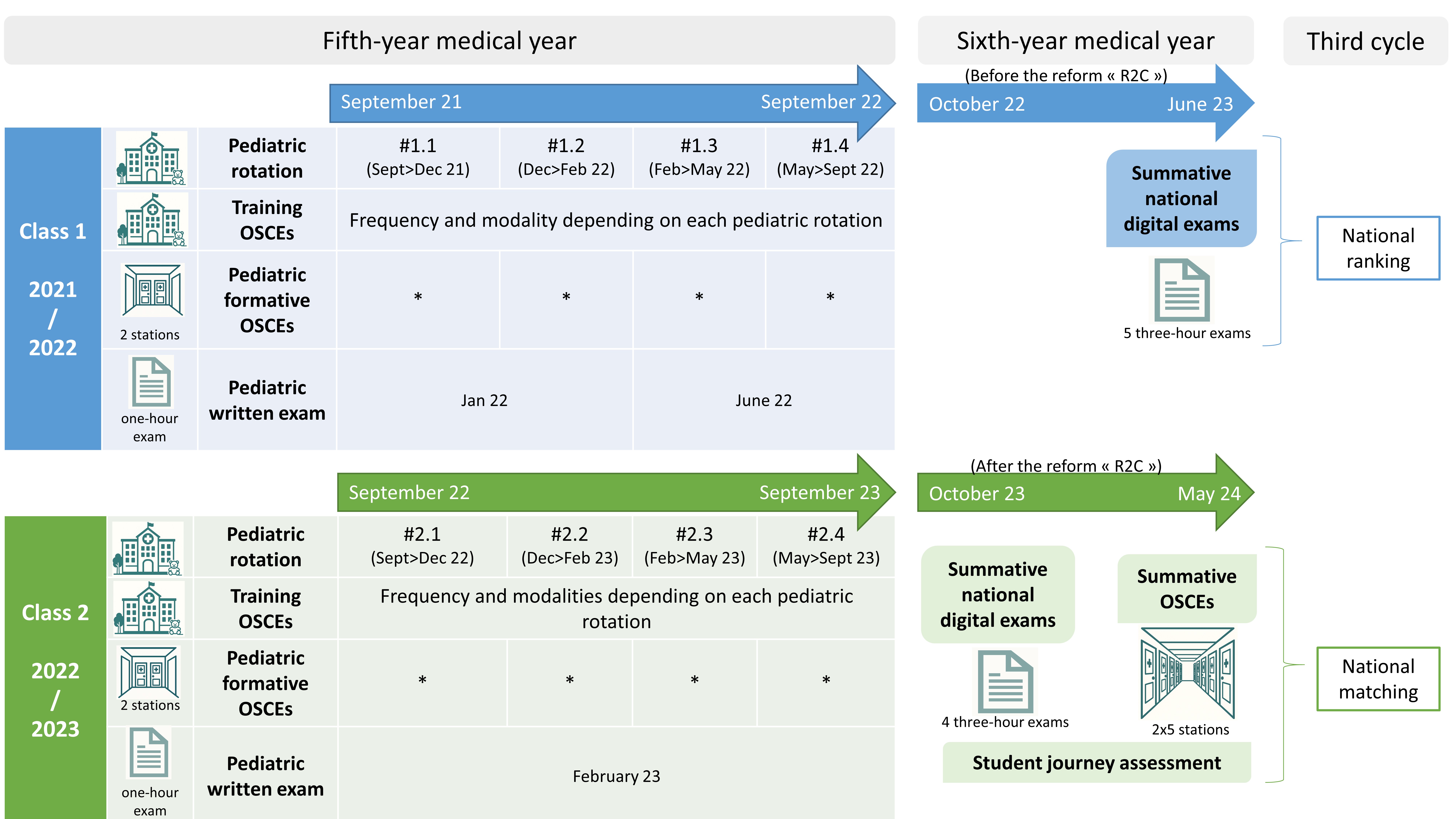

Supplement: Supplementary file 2 — Supplementary Material 2. Supplementary figure: chronology of formative and summative examinations by class, according to the second-cycle reform (R2C). [file 12909_2026_8999_MOESM2_ESM.jpg]
